# Recent advances in the multicomponent synthesis of heterocycles using tetronic acid

**DOI:** 10.1039/d3ra02505e

**Published:** 2023-06-02

**Authors:** Ramin Javahershenas, Sahand Nikzat

**Affiliations:** a Department of Organic Chemistry, Faculty of Chemistry, Urmia University Urmia Iran jshbco@yahoo.com; b Chemical Physics Theory Group, Department of Chemistry, University of Toronto Toronto M5S 3H6 ON M5S 3H6 Canada sahand.nikzat@mail.utoronto.ca

## Abstract

Tetronic acid, a versatile synthon, has been extensively investigated by numerous researchers in synthetic chemistry due to its crucial role in synthesizing heterocycles which makes this compound particularly advantageous in both pharmaceutical and biological fields. Various heterocycles can be synthesized using it as a precursor *via* multicomponent reactions (MCRs). Dicarbonyl groups can be considered the building blocks and key structural motifs of a wide range of natural compounds, which may contain different functional groups in the synthesis of heterocyclic frameworks. This review covers the literature from 2017 to 2022, and it encompasses the different one-pot protocols for synthesizing a variety of heterocyclic molecules.

## Abstract 2

Tetronic acid, a versatile synthon, has been extensively investigated by numerous researchers in synthetic chemistry due to its crucial role in synthesizing heterocycles which makes this compound particularly advantageous in both pharmaceutical and biological fields.

## Graphical abstract

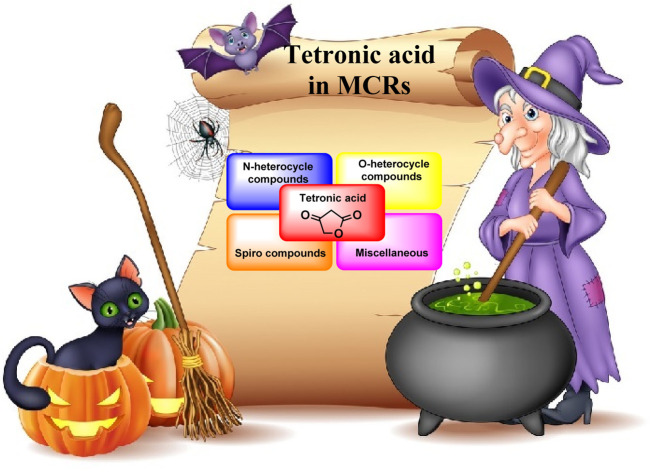

## 1. Introduction

Given the paramount significance of human health and hygiene, researchers in medicinal chemistry continue to pursue the synthesis of bioactive molecules with unrelenting vigor. These efforts aim to develop effective treatments for various diseases and to restrain the transmission of infectious diseases.^1,2^ The design and preparation of cost-effective drugs that establish high efficacy with minimal side effects is a vital objective for researchers in this field, to combat a variety of diseases. To achieve this goal, considerable effort has been dedicated to the synthesis and screening of candidate molecules to ensure low toxicity and side effects.^3,4^ Furthermore, novel drug discovery has been transformed by this emerging paradigm. The main backbone and structure of most organic compounds made so far are composed of heterocycles.^5^

**Fig. 1:**
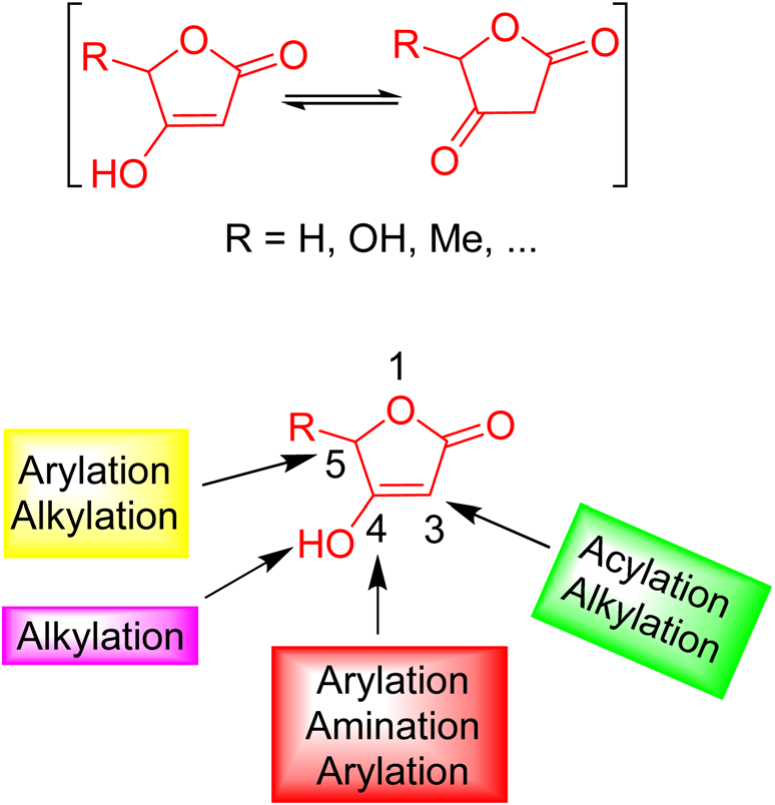
Structure and reactivity of tetronic acids (4-hydroxy-[5*H*]furan-2-one).

This emerging paradigm has substantially revolutionized the field of novel drug discovery. Notably, heterocycles are the primary backbone and structure of numerous synthesized organic compounds. In the context of versatile heterocyclic motifs, tetronic acids (4-hydroxy-[5*H*]furan-2-one) have been proven to be a versatile and convenient building block.^6,7^ This unique structural framework serves a pivotal role in organic synthesis owing to its relative presence in natural products. Consequently, their chemical and medicinal properties gained substantial interest. The overall structure and reactivity of tetronic acids along with nucleophilic and electrophilic active centers are shown in Fig. 1.^8,9^

The biological and pharmaceutical activities of these compounds can distinguish them as noteworthy classes of chemical substances. For instance, nitrogen and oxygen based heterocyclic frameworks can be regarded as a distinguished category due to their extensive abundance in natural compounds.^10^

Recently, the focus of pharmaceutical chemists has progressively shifted towards tetronic acids and their derivatives, prompted by the diverse therapeutic properties these compounds demonstrate, and this interest has catalyzed the development of innovative methodologies.

**Fig. 2:**
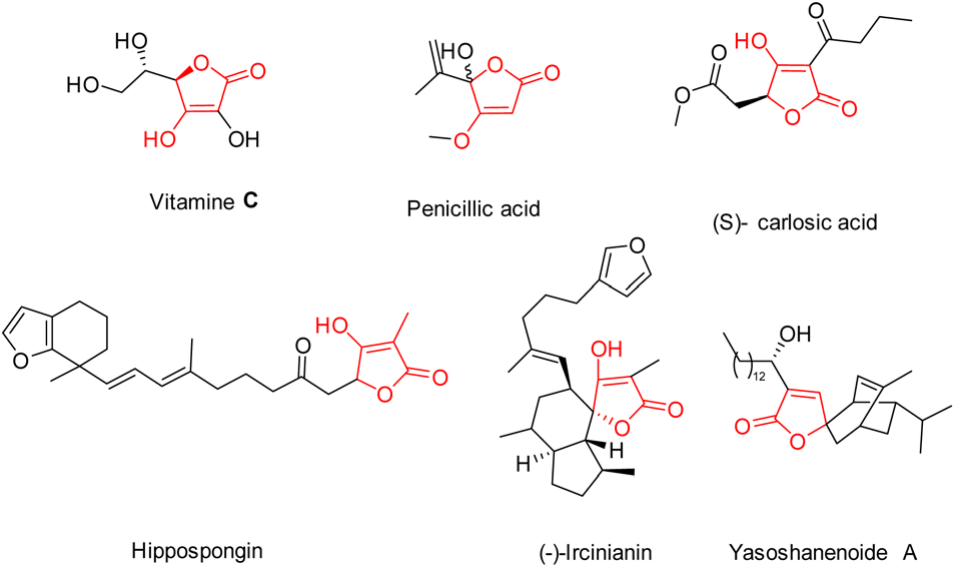
The structure of several natural products contains the framework of tetronic acids.

Within the class of natural five-membered heterocycles featuring *O*-substituted derivatives, tetronic acids (4-hydroxy-[5*H*]furan-2-ones) stand out owing to their profusion. A substantial number of natural compounds that stand for this particular heterocyclic structure have been identified, as illustrated in Fig. 2. The most notable instances of these compounds are penicillin and ascorbic acid (vitamin C).^11,12^

There is noteworthy attention on tetronic acid derivatives as a result of their diverse biological and metabolic activities. These include functioning as anti-inflammatory,^13^ anticancer,^14^ anti-HIV-1 protease,^15^ antiepileptic,^16^ antibiotic,^17,18^ antifungal,^19^ anticoagulant,^20^ antibacterial,^21^ analgesic,^22^ and insecticidal agents.^23^

A multicomponent reaction involves assembling products from multiple starting materials in a single-step protocol (MCRs). The utility of MCRs in facilitating the synthesis of diverse chemical structures has been recognized for over a century. Drug companies may take advantage of multicomponent reactions (MCRs). These reactions can be utilized to synthesize highly functionalized, biologically relevant natural and active molecules, including polycyclic structures.

Moreover, MCRs can be employed to boost various processes within pharmaceutical synthesis. Organic chemists primarily employ multicomponent reactions in the design and preparation of intricate, polycyclic molecules. The appeal of these reactions lies in their ability to minimize by-product formation, optimize energy consumption, reduce reaction times, maximize yields, and enhance selectivity. As such, the amalgamation of one-pot multicomponent strategies with diverse methodologies expedites the synthesis of biologically pertinent heterocycles, a cornerstone of 21st-century organic chemistry.^24–28^

In a bid to extend research in the field of heterocyclic scaffold synthesis through multicomponent reactions,^29–34^ this work emphasizes the recurrent use of tetronic acid as an initial substrate in the synthesis of these compounds. The objective of this study is to examine the recent advancements in multicomponent reactions involving tetronic acid, a highly versatile moiety in heterocyclic chemistry, as a central structural motif for heterocyclic compounds. This review encompasses literature reports from the period 2017–2022.

Synthetic molecules can be categorized into four types according to their structural characteristics, which assist in their study. These types are organized based on the structural framework of the final product, including N-heterocycle compounds, O-heterocycle compounds, spiro compounds, and miscellaneous compounds. This classification method provides an easier and more convenient approach to examining these molecules.

## 2. Synthesis of N-heterocycle compounds

### 2.1. Furo[3′,4′:5,6]pyrido[2,3-*c*]carbazole

**Scheme 1:**
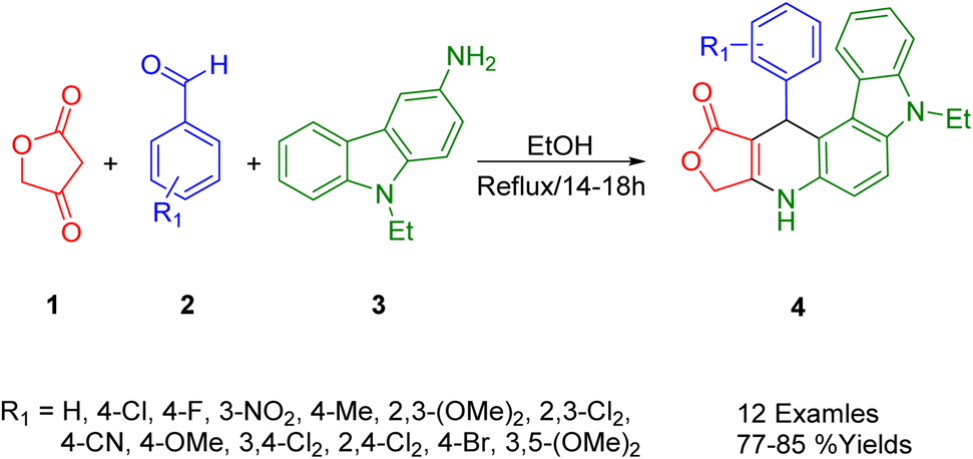
Synthesis of pyrido[2,3-*c*]carbazole derivatives.

Using tetronic acid (1), substituted benzaldehyde (2), and 9-ethyl-9*H*-carbazol-3-amine (3), Wang *et al.*, performed a one-pot catalyst-free multicomponent reaction and obtained a series of furo[3′,4′:5,6]pyrido[2,3-*c*]carbazol-1-one derivatives (4) in ethanol (Scheme 1). In this protocol, pyrido[2,3-*c*]carbazole derivatives form an uncatalyzed reaction that occurs under mild conditions, thus offering the advantage of mild reaction conditions.^35^

### 2.2. Furo[3′,4′:5,6]pyrido[2,3-*d*]pyrimidine

**Scheme 2:**
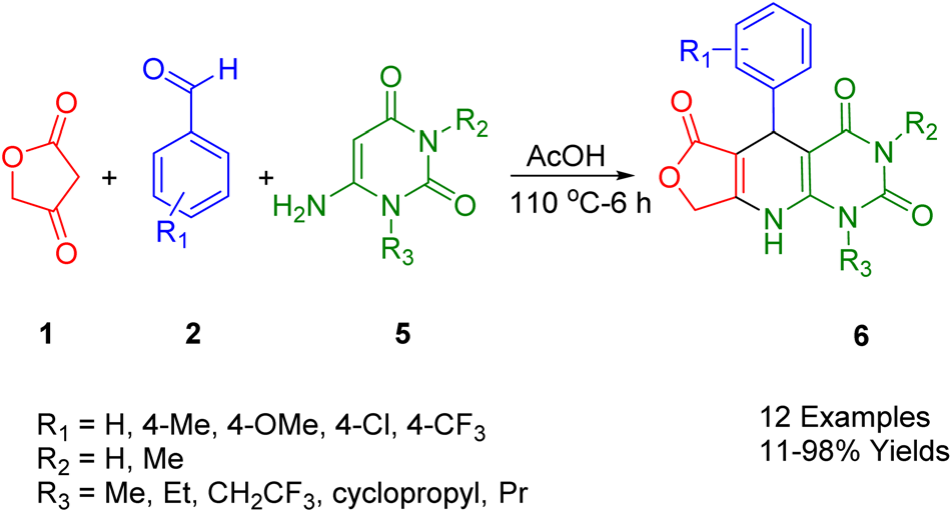
Synthesis of dihydropyridopyrimidines.

Pomerantz *et al.*, described a novel synthesis of pyrido[2,3-*d*]pyrimidin (6), utilizing simple and readily available raw materials to generate a series of pyrido[2,3-*d*]pyrimidin (6) in yields ranging from low to high (Scheme 2). The synthesis of dihydropyridine was achieved through a three-component condensation of tetronic acid (1), various substituents of benzaldehyde (2), and uracil (5) as starting materials, yielding the corresponding desired products. The methodology to prepare various pyrido[2,3-*d*]pyrimidine proposes numerous advantages, such as brief reaction times, methodological simplicity, and comfortable reaction conditions. Moreover, the application of a straightforward washing method sufficed for the isolation of pure compounds, circumventing the necessity for rigorous chromatographic techniques.^36^

### 2.3. Dihydrodibenzo[*f*,*h*]furo[3,4-*b*]quinolone

**Scheme 3:**
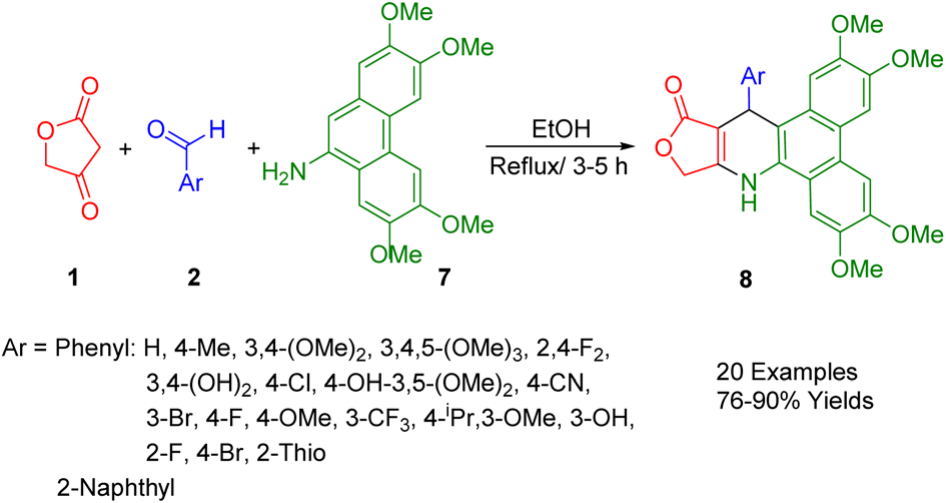
Synthesis of dihydropyridophenanthrene compounds.

Kumar *et al.*, developed a simple and convenient method of synthesizing 4-*aza*-2,3-dihydropyridophenanthren derivatives (8) with reasonable yields through domino reactions of tetronic acid (1), aryl aldehydes (2), and 2,3,6,7-tetramethoxyphenanthren-9-amine (7), all of which can be used in refluxing ethanol (Scheme 3).^37^

Initially, the formation of benzylidene-2,4-furanone involves a reaction between tetronic acid (1) and aryl aldehydes (2) through Knoevenagel condensation. Following Michael's addition of the amino group, a water molecule is eliminated through a cyclization process, yielding the final product.

A comprehensive study has investigated a broad spectrum of synthetic compounds for their anticancer and antitumor properties. The types of cancers tested in this study encompassed cells from breast, colon, lung, gastric, prostate, and cervical cancers.

### 2.4. Furo[3,4-*b*]pyrazoloquinoline

**Scheme 4:**
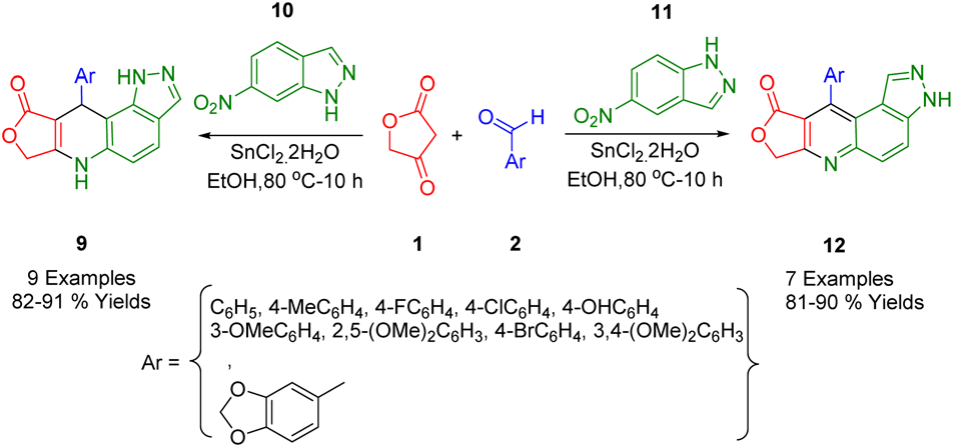
Synthesis of pyrazolo[3,4-*f*]quinoline and pyrazolo[4,3-*f*]quinoline compounds.

The Rong group developed a new approach that combines *in situ* reduction and annulation to synthesize pyrazolo[3,4-*f*]quinoline (12) and pyrazolo[4,3-*f*]quinoline derivatives (15). These were obtained by the cycloaddition of 5-nitroindazoles (13) in a 1,3-dipolar form, and 6-nitroindazoles (14) using different aromatic aldehydes (2), along with tetronic acid (1) in the presence of SnCl_2_·2H_2_O (Scheme 4). This method outperforms traditional synthetic methods due to several advantages, including stable reagents, simplicity, easy access to inexpensive raw materials, and high yields. In this highly efficient *in situ* reduction method, SnCl_2_·2H_2_O is used to simultaneously reduce nitro compounds and form a ring. Furthermore, this process provides a suitable alternative strategy for synthesizing scaffolds for other applications.^38^

### 2.5. Dihydrofuro[3′,4′:5,6]pyrido[3,2-*f*]quinoxaline

**Scheme 5:**
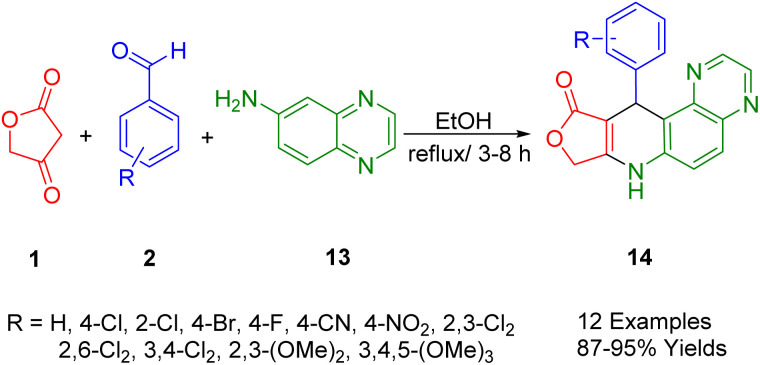
Synthesis of furo[3′,4′:5,6]pyrido[3,2-*f*]quinoxalin-10(7*H*)-one derivatives.

Wang *et al.*, have reportedly conducted a successful one-pot multicomponent condensation of tetronic acid (1), quinoxaline-6-amine (16), and aryl aldehydes (2) to synthesize the dihydrofuro[3′,4′:5,6]pyrido[3,2-*f*]quinoxalin-10(7*H*)-one derivative (17) under reflux conditions in ethanol (Scheme 5). The structural reliability of the synthetic compound 11-(4-nitrophenyl)-8,11-dihydrofuro[3′,4′:5,6]pyrido[3,2-*f*]quinoxalin-10(7*H*)-one was confirmed *via* X-ray diffraction analysis. This simple and practical method paves the way for the synthesis of fused tetracyclic heterocycles without the need for heavy catalysts. Utilizing fractions of tetracyclic amines, pyridine, and furan, this method offers the advantages of operational simplicity and avoids the necessity for separate intermediates in the reaction.^39^

### 2.6. Dihydrofuro[3,4-*b*]quinoline

**Scheme 6:**
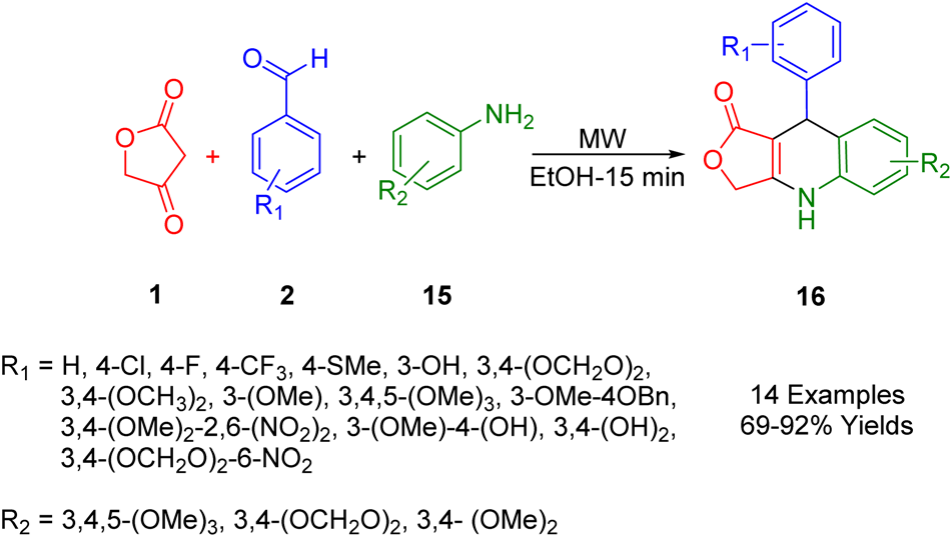
Synthesis of dihydroquinoline lactone derivatives.

Laurentiz *et al.*, have developed a straightforward and efficient method for synthesizing dihydroquinoline lactone derivatives (19). This process, which yields good to high results, involves the reaction of tetronic acids (1), aryl anilines (18), and aromatic aldehydes (2) in refluxing ethanol (Scheme 6).^40^

In subsequent studies, synthetic compounds were tested for antibacterial activity under laboratory conditions against various bacterial strains, including *Streptococcus mitis*, *Prevotella nigrescens*, *Streptococcus sanguinis*, and *Porphyromonas gingivalis*. In addition, these compounds were screened for their effectiveness against *Mycobacterium avium*, *Mycobacterium tuberculosis*, and *Mycobacterium kansas*. An analysis of the results revealed that these compounds were particularly effective against Gram-negative bacteria. The influence of side groups on the activity of these compounds was also assessed. Findings indicated that the presence of a methylenedioxy group on the dihydroquinoline ring and a nitro group on the structure-activity ring enhanced the antibacterial activity of the derivatives.

### 2.7. Diphenyl-1,4,5,7-tetrahydrofuro[3,4-*b*]pyridine

**Scheme 7:**
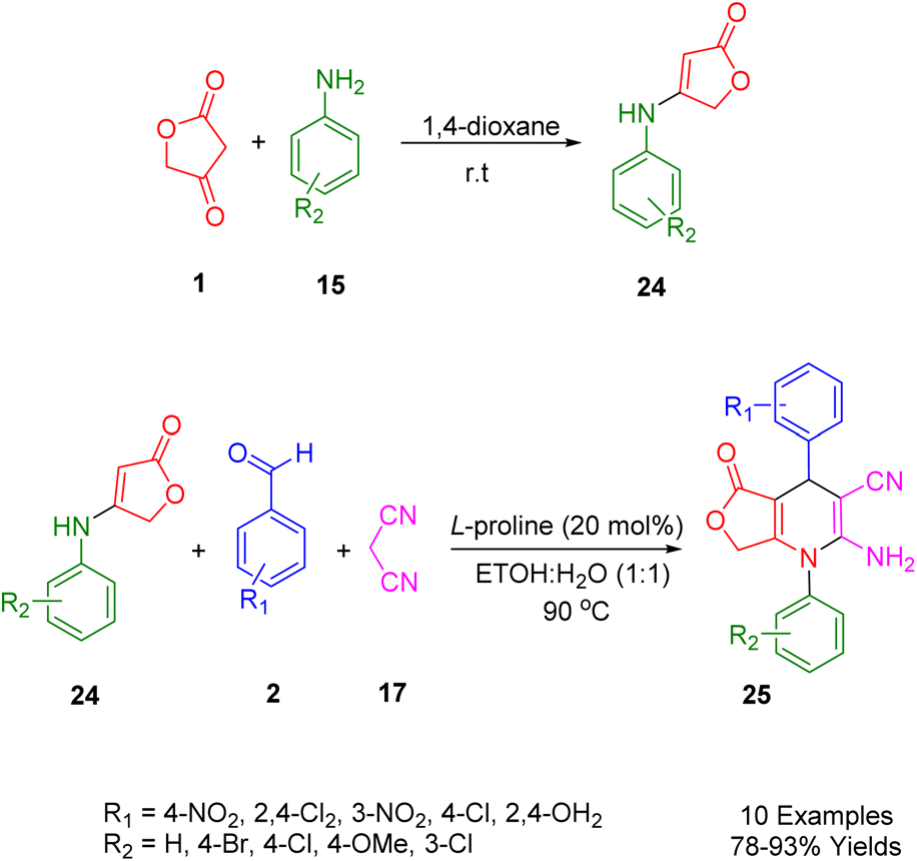
Synthesis of 1,4-DHPs compounds.

Moradi *et al.*, focused on an eco-friendly and mild synthesis of novel 1,4-dihydropyridines scaffolds (30) using a multicomponent reaction. The starting material, anilolactone (29), was obtained from the reaction between tetronic acid (1) and substituted anilines (18). This was then combined with malononitrile (20) and substituted benzaldehydes (2) under optimized conditions (90 °C in 10 mL of EtOH/H_2_O (1 : 1) as solvent). The use of 20 mol% of l-proline as an effective homogeneous catalyst led to the production of the corresponding products in high yields (Scheme 7).^41^

To optimize the synthetic method of the above-mentioned heterocycle structures, a comparative study of a domino, one-pot reaction involving 4-nitrobenzaldehyde (2), malononitrile (20), and anilolactones (29) was conducted. An experiment was performed in aqueous ethanol, combining 4-nitrobenzaldehyde (2), malononitrile (20), tetronic acid (1), and three types of aniline (18) in a one-pot reaction using l-proline as a catalyst. The efficacy of this method (82–93%) was significantly higher than that of the four-component reactions (47–71%), demonstrating the superiority of the one-pot three-component approach.

**Scheme 8:**
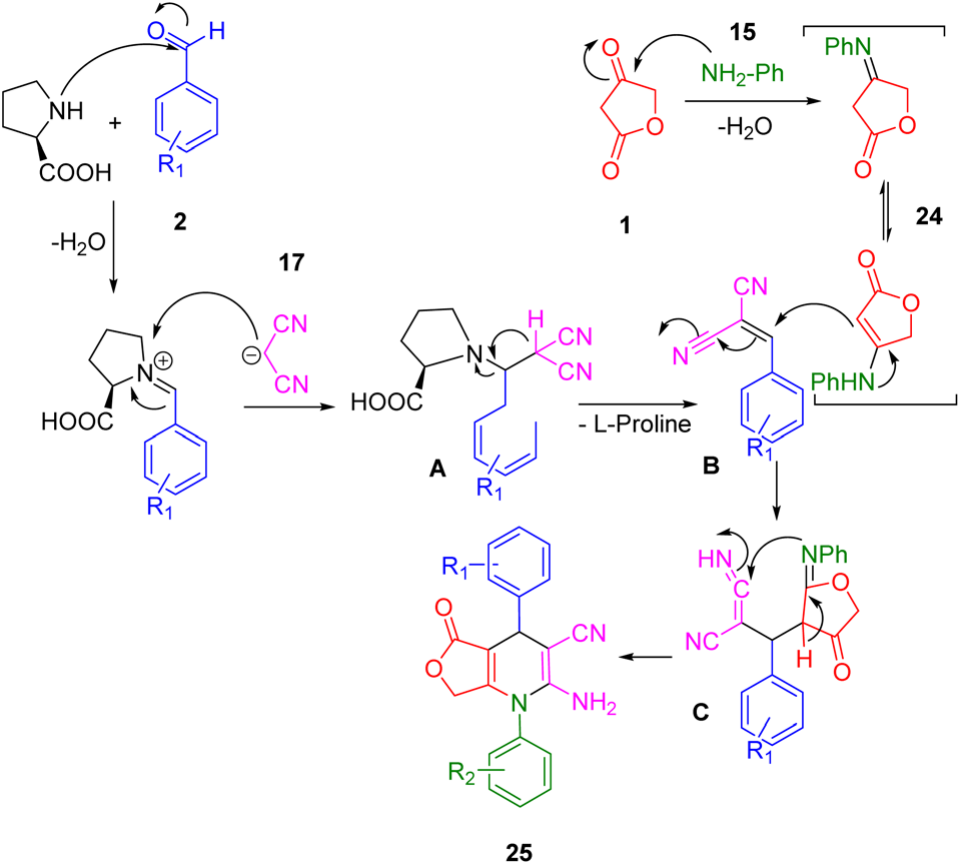
The proposed mechanism for the three-component synthesis of 1,4-DHPs.

The mechanism of the reaction was demonstrated through the interaction between anilolactone (29), benzaldehyde (2), and malononitrile (20), as depicted in Scheme 8. Knoevenagel condensation of benzaldehyde and malononitrile in the presence of l-proline initially generated intermediate A, which was then converted into intermediate B upon removal of the catalyst. Simultaneously, the condensation of tetronic acid (1) and aniline (18) produced anilolactone (29) and its enamine. Subsequently, an intermolecular Michael addition between intermediate B and anilolactone (29) yielded intermediate C. This was further transformed into its enamine form. The desired product (30) was finally obtained through cyclocondensation and tautomerization, facilitated by the intramolecular nucleophilic attack of the enamine N atom on the cyano group.

### 2.8. Furo[3,4-*b*]naphtho[2,3-*f*]quinoline

**Scheme 9:**
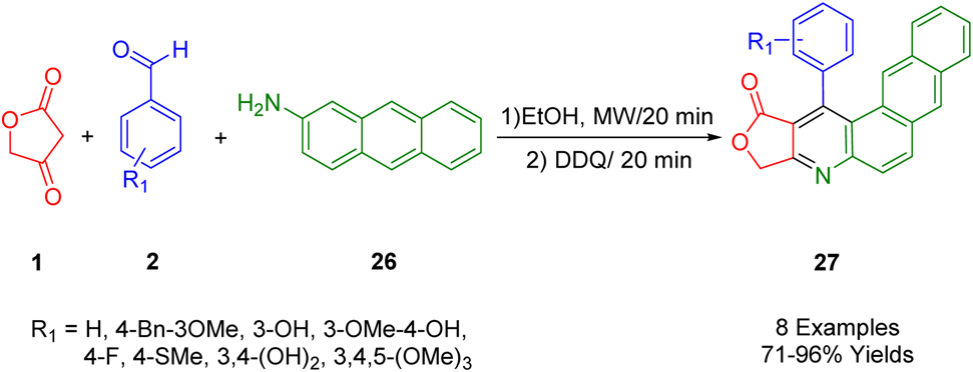
Synthesis of naphthoquinoline derivatives.

de Laurentiz *et al.*, reported the use of tetronic acid (1), 2-aminoanthracene (31), and various aromatic aldehydes (2) as starting materials in a three-component reaction (Scheme 9). This reaction, upon the addition of 2,3-dichloro-5,6-dicyano-1,4-benzoquinone (DDQ), resulted in the generation of naphtho[2,3-*f*]quinoline lactone compounds (32) with yields ranging from satisfactory to high.^42^

The UV-vis and fluorescence spectra, as well as the optical absorption and emission properties of these synthetic derivatives, were studied in various solvents and at different pH levels. Emission bands in the range of 453–517 nm were observed for these derivatives, along with a bathochromic shift (597 nm) attributed to excited-state intramolecular proton transfer (ESIPT) in the case of a compound (R_1_ = 3,4-(OH)_2_). This compound showcased this catalysis. Although its luminescence properties were still preliminary, the results indicated its potential use as a chemical sensor across different solvents and pH levels.

### 2.9. Dihydrobenzo[*g*]furo[3,4-*b*]quinoline

**Scheme 10:**
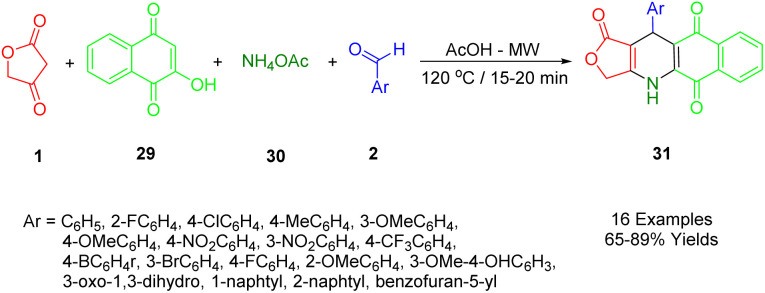
Synthesis of aryl-4,11-dihydrobenzo[*g*]furo[3,4-*b*]quinoline-1,5,10(3*H*)-triones.

Nguyen *et al.*, applied microwave-assisted methods to prepare novel aryl-4,11-dihydrobenzo[*g*]furo[3,4-*b*]quinoline-1,5,10(3*H*)-trione derivatives (31) in glacial acetic acid at 120 °C. They reported a modification of the domino reaction with tetronic acid (1), 2-hydroxy-1,4-naphthoquinone (29), benzaldehydes (2), and ammonium acetate (30) in glacial acetic acid using a microwave reaction. Yields ranged from good to moderate (Scheme 10).^43^

The cytotoxicity activities of all synthesized compounds were extensively analyzed against MCF7, KB, Lu1, HepG2, and non-cancerous Hek-293 cell lines. A study was conducted on the potential value of these compounds in developing potential anticancer agents by the fact that some of the synthetic compounds displayed highly potent inhibitory activities with IC50s > 40 nM against SK-Lu-1 and HepG2 cell lines, and for non-cancerous Hek293 cell lines had less toxicity.

**Scheme 11:**
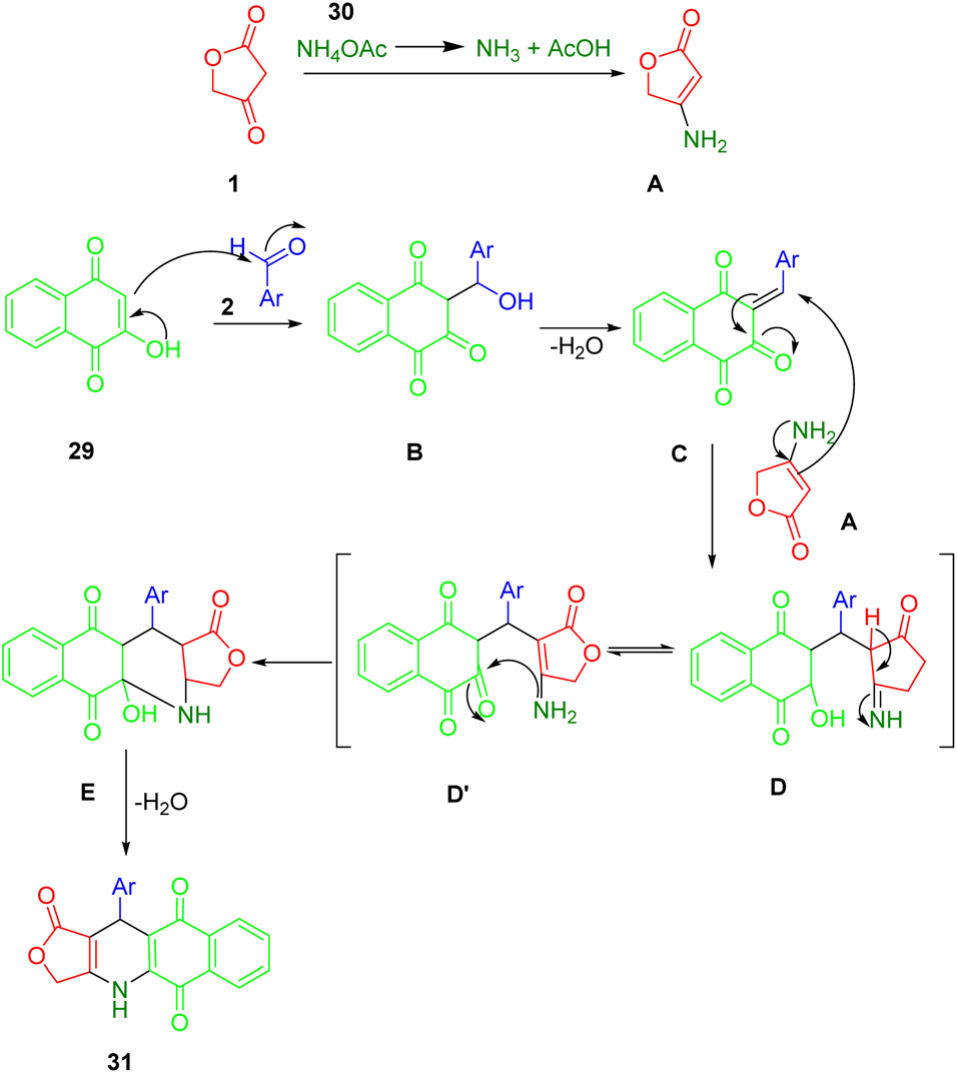
A plausible mechanism for the synthesis of podophyllotoxin-naphthoquinones.

The preparation process for a series of dihydrobenzo[*g*]furo[3,4-*b*]quinoline-1,5,10(3*H*)-triones (36) involves a plausible and acceptable four-component reaction mechanism. Initially, 1,4-naphthoquinone (34) undergoes Knoevenagel condensation with substituted benzaldehydes (2), followed by the loss of water molecules, to afford intermediates C. Then, Michael's addition occurs within intermediate C, followed by intramolecular cyclization and another round of water molecule loss to yield the final product (36), as demonstrated in Scheme 11.

**Scheme 12:**
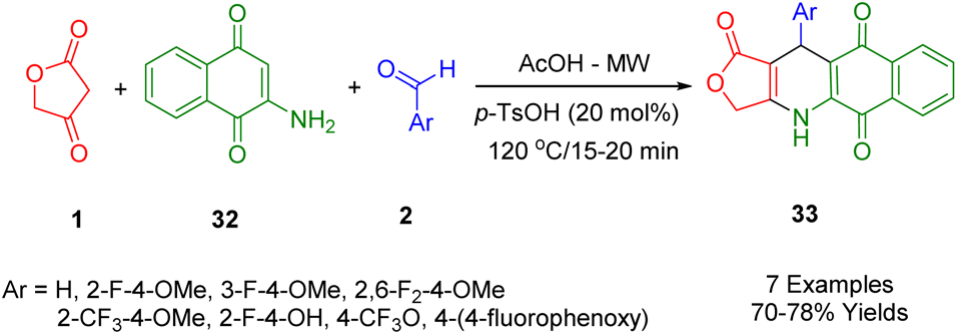
Synthesis of novel naphthoquinone-fused podophyllotoxins.

Novel bioactive naphthoquinone-fused podophyllotoxins with fluoro- and trifluoromethyl scaffolds have been synthesized by Van Kiem *et al.*, and produced satisfactory yields with the use of MW by the one-pot multicomponent condensation of 2-amino-1,4-naphthoquinone (32), tetronic acid (1), and fluorinated aryl aldehydes (2) in glacial acetic acid at 120 °C (Scheme 12). Additionally, some of the products studied were examined for their anticancer activity, and the results showed that some had promising anticancer properties against human embryonic kidney cells (Hek-293), human hepatocellular carcinoma cells [HepG2], breast carcinoma cells [MCF7], lung cancer cells [A549], and human cancer cell lines (the MTT assay was used to test human carcinoma [KB]).^44^

**Scheme 13:**
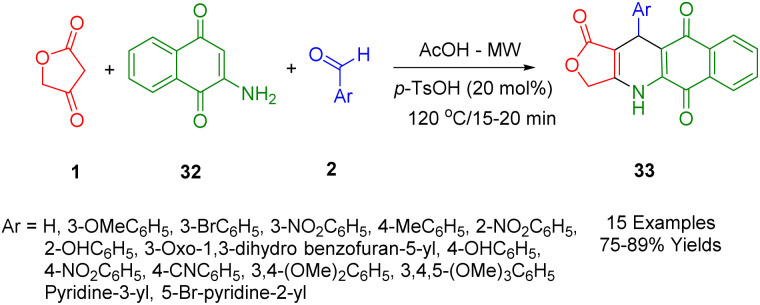
Synthesis of podophyllotoxin-naphthoquinone.

A new class of agents with potential antitumor activity, known as podophyllotoxin-naphthoquinone derivatives (33), are derived from 2-aminonaphthalene-1,4-dione (32) in Scheme 13 in the same procedures as described.^45^

In a separate study, Van Nguyen *et al.*, showed that microwave-assisted three-component reactions can be used to synthesize podophyllotoxin-naphthoquinone compounds (33) using the stepwise thermal reaction.^46^ The cytotoxicity profile of each synthetic compound was tested against four cancerous cell lines (MCF7, A549, KB, and HepG2) as well as against a non-cancerous cell line, Hek-293. In particular, it was found that treatment of SK-LU-1 cells with compounds (Ar = 3-OMeC_6_H_5_ and 3-BrC_6_H_5_) resulted in an arrest of the G2/M phase of the cell cycle, activation of caspase-3/7, and an increase in apoptosis. Moreover, the results of a molecular binding study of these compounds showed that they had an outstanding interaction with the residues in the tubulin colchicine binding site.

**Scheme 14:**
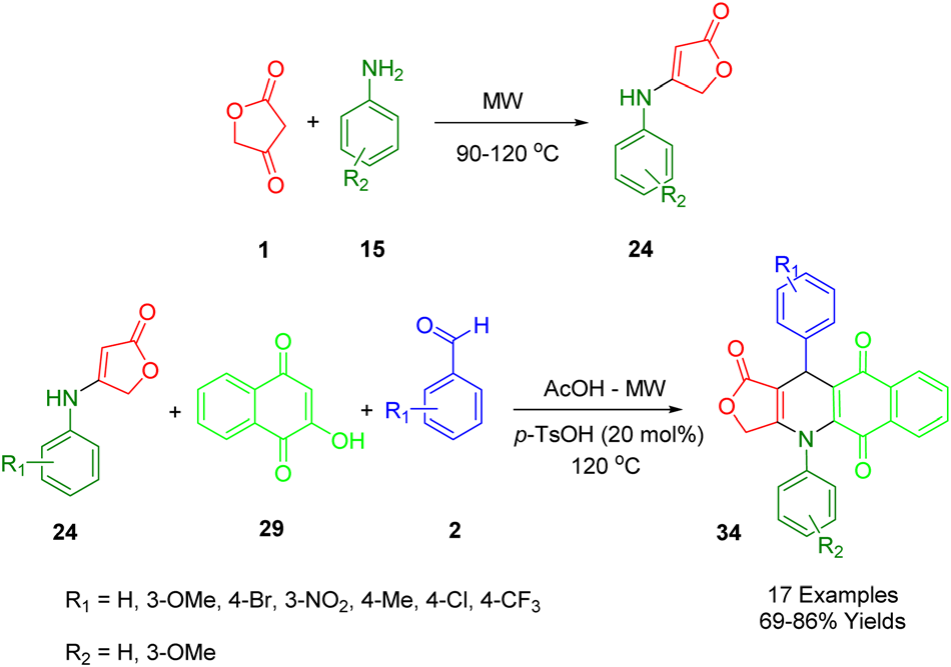
Synthesis of furo[3,4-*b*]quinoline-1,5,10(3*H*)-triones.

A novel, convenient, and significant domino synthetic protocol has been described by Van Nguyen and co-workers, which can be used to synthesize healthy levels of 4,11-diaryl-4,11-dihydrobenzo[*g*]furo[3,4-*b*]quinoline-1,5,10(3*H*)-trione (34) in a high-yield (Scheme 14). In order to obtain the desired starting material, under microwave irradiation multicomponent protocol of 1,4-naphthoquinone (28), aryl aldehydes (2), and anilolactone 4-((3-methoxyphenyl)amino)furan-2(5*H*)-one (23) was employed, utilizing the reaction between tetronic acid (1) and substituted anilines (15).^46^

The model reaction was used for the preparation of furo[3,4-*b*]quinoline-1,5,10(3*H*)-trione derivatives as a way to demonstrate the convenient strategy for the preparation of furo[3,4-*b*]quinoline-1,5,10(3*H*)-trione derivatives (34). In order to produce the final product, some sequential multi-step reactions must be carried out including Knoevenagel condensation, Michael's addition, the shift of hydrogen, intramolecular cyclocondensation reaction, and finally through the loss of water molecules. A comprehensive evaluation of the cytotoxicity profile of all products (Hek-293) against four cancer cell lines (MCF7, A549, HepG2, and KB) as well as human embryonic kidney cell lines has been conducted.

### 2.10. Tetrahydro-1*H*-benzo[*b*]furo[3,4-*e*][1,4]diazepine

**Scheme 15:**
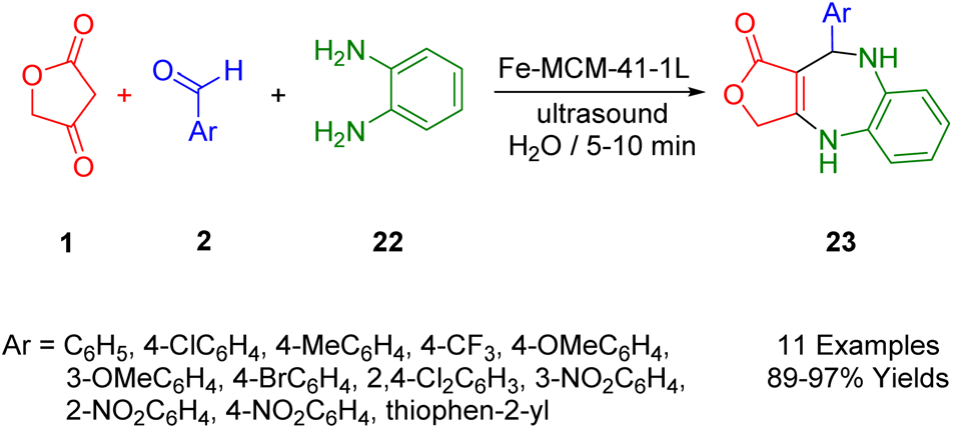
Sonosynthesis of benzodiazepines.

Naeimi *et al.*, presented a clean, efficient, and straightforward procedure for the ultrasound-assisted multicomponent synthesis of biologically relevant benzodiazepine molecules (26) with tetracyclic frameworks. The process involves reactions between tetronic acid (1), aldehydes (2), and *o*-phenyldiamine (25), facilitated by molecular ionic liquid supported on Fe-MCM-41-IL nanocomposites in H_2_O (Scheme 15). This approach enables the rapid production of benzodiazepine structures using ultrasound, offering the advantages of speed, cleanliness, and experimental convenience. The catalyst, which can be easily applied using an external magnet, demonstrated consistent activity, suggesting potential for repeated use without significant degradation in performance.^47^

## 3. Synthesis of O-heterocycle compounds

### 3.1. 4*H*-Furo[3,4-*b*]pyran

**Scheme 16:**
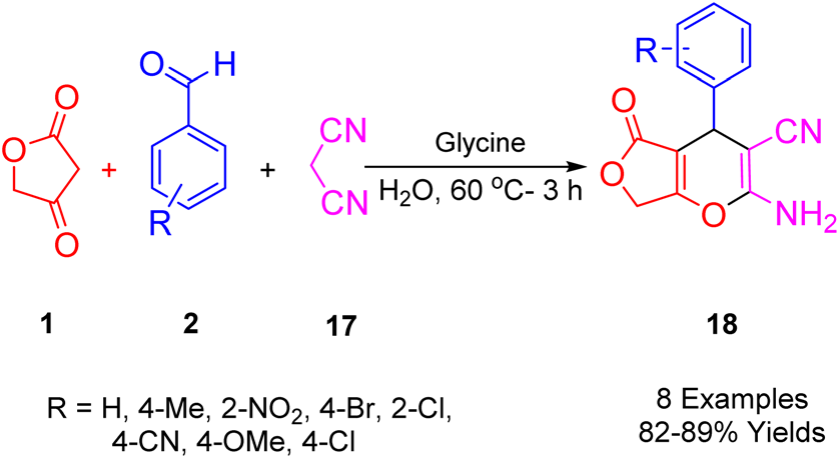
Synthesis of 4*H*-furo[3,4-*b*]pyran derivatives.

A novel series of polyfunctionalized, biologically significant 4*H*-furo[3,4-*b*]pyran scaffolds (21) was synthesized by Singh *et al.*, (Scheme 16). This was achieved *via* a facile, glycine-catalyzed, one-pot multicomponent reaction. The process efficiently combined substituted benzaldehyde (2), malononitrile (20), and tetronic acid (1) in water at 60 °C to yield the desired bioactive furo[3,4-*b*]pyran derivatives. This method is characterized by its benign environmental reaction conditions, high atomic economy, operational simplicity, cost-effectiveness, environmental compatibility, non-corrosiveness, and excellent performance. Moreover, it utilizes glycine as a cheap, readily available, and recoverable organocatalyst, eliminating the need for expensive metal catalysts.^48^

**Scheme 17:**
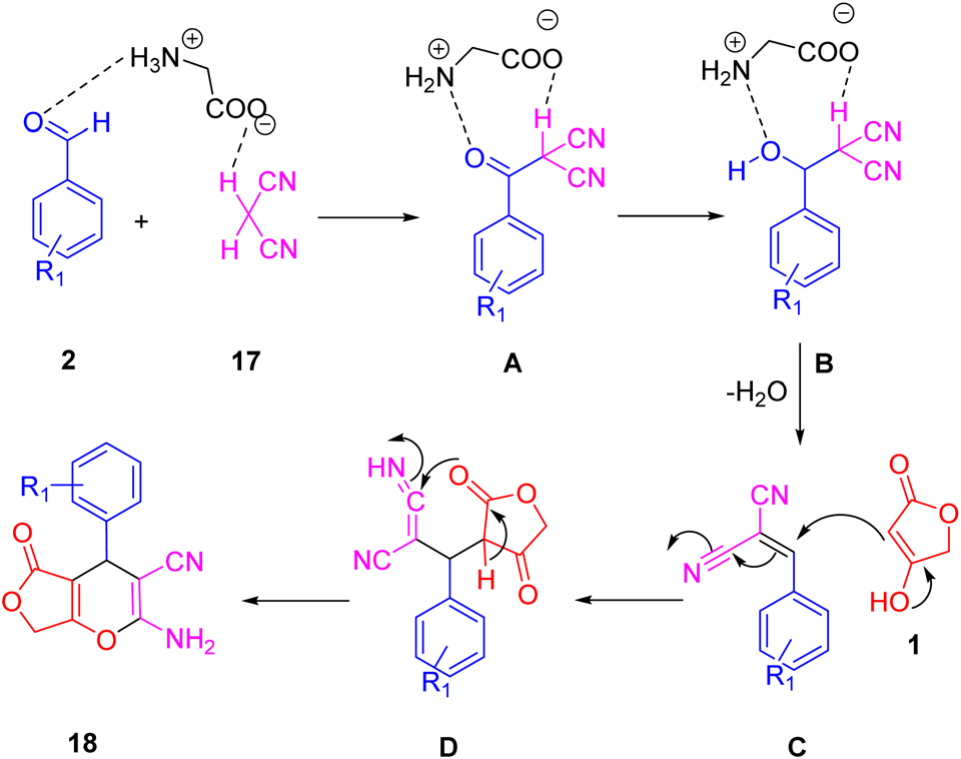
A plausible mechanism for the synthesis of polyfunctionalized 4*H*-furo[3,4-*b*]pyran.

Scheme 17 illustrates the proposed mechanism for synthesizing the desired heterocycles. Glycine effectively facilitates the Knoevenagel condensation of malononitrile (20) with an aldehyde (2). Subsequently, a Michael-type addition of tetronic acid (1) to the resulting cyanoolefin intermediate (B) occurs. The electron pair on the oxygen atom then initiates an attack, leading to the formation of the proposed furopyran compound (21).

### 3.2. Furo[3,4-*b*]chromene-1,5(6*H*)-dione

**Scheme 18:**
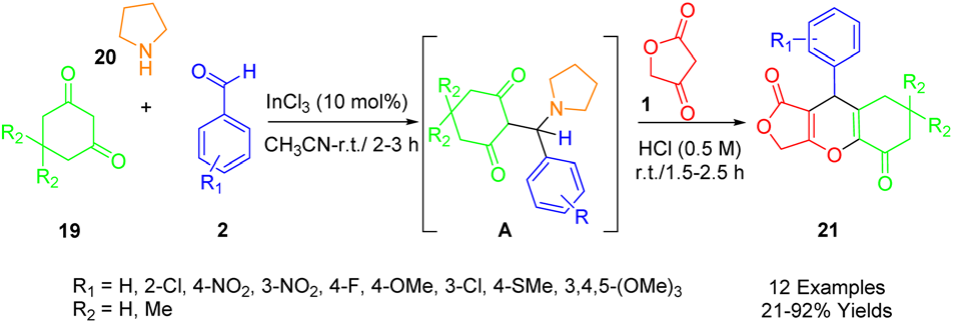
Synthesis of furo[3,4-*b*]chromene-1,5(6*H*)-dione derivatives.

Roche *et al.*, outlined an efficient and rapid one-pot synthesis of novel furo[3,4-*b*]chromene-1,5(6*H*)-dione derivatives (24) (Scheme 11). Given their medicinal chemistry applications, 4*H*-pyrans (4*H*-pys) are favored heterocyclic moieties among chemists (Scheme 18). This report presents a one-pot multicomponent reaction for the preparation of polyfunctionalized 4*H*-pys (24). This reaction involves pyrrolidine (23), various substituted aldehydes (2), dimedone (22), and tetronic acid (1), and uses InCl_3_ (10 mol%) as a catalyst under solvent-free conditions. The synthesis of these compounds employs a two-step protocol. Initially, the intermediate A is produced by the reaction of aromatic aldehyde (2), pyrrolidine (23), and dimedone (22). This is followed by the *in situ* reaction of tetronic acid (1) with intermediate A, without separation, to obtain the fused-tricyclic target compounds (24).^49^

**Scheme 19:**
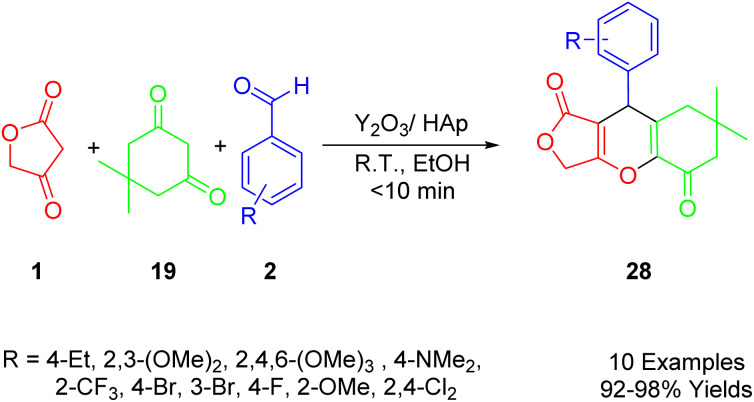
Synthesis of novel furo[3,4-*b*]chromenes.

Maddila *et al.*, developed an efficient, environmentally friendly, multi-component method for the synthesis of furo[3,4-*b*]chromenes (33) using yttria-doped hydroxyapatite (Y_2_O_3_/HAp) as a heterogeneous and recyclable catalyst (Scheme 19). This synthetic method involves a sequential Knoevenagel condensation, which is a type of 1,3-dipolar reaction. The reaction takes place at room temperature in ethanol, employing tetronic acid (1), substituted aldehydes (2), and dimedone (22) to yield the desired compounds (33) with excellent efficiency (91–98%). The approach aligns with principles of green chemistry, utilizing environmentally friendly, non-toxic solvents, and a simple method. It also boasts a short reaction time, removes the need for column chromatography, and enables catalyst reuse.^50^

## 4. Spiro compounds

### 4.1. Spirooxindoles

**Scheme 20:**
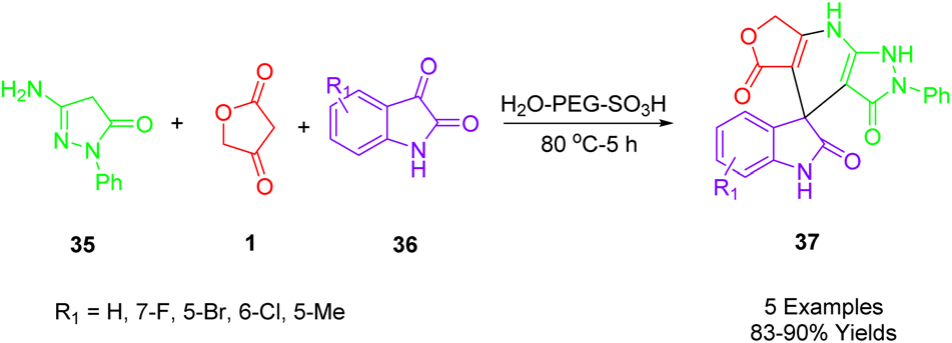
Synthesis of spiro[furo[3,4-*b*]pyrazolo[4,3-*e*]pyridine-4,3′-indoline]-2′,3,5(7*H*,8*H*)-triones.

Rong and colleagues described a simple, green, and efficient one-pot reaction to synthesize a diverse spiro[furo[3,4-*b*]pyrazolo[4,3-*e*]pyridine-4,3′-indoline]-2′,3,5(7*H*,8*H*)-trione (37) using three components (Scheme 20). In an aqueous medium, 3-amino-1-phenyl-2-pyrazoline-5-one (35), isatin (36), and tetronic acid (1) as a substrate are combined with high yields in the presence of PEG-SO_3_H. An aqueous medium at 80 °C was used for the catalysis of this reaction, which was catalyzed by PEG-SO_3_H. The recyclability of the catalyst has been reported as high as five times.^51^

**Scheme 21:**
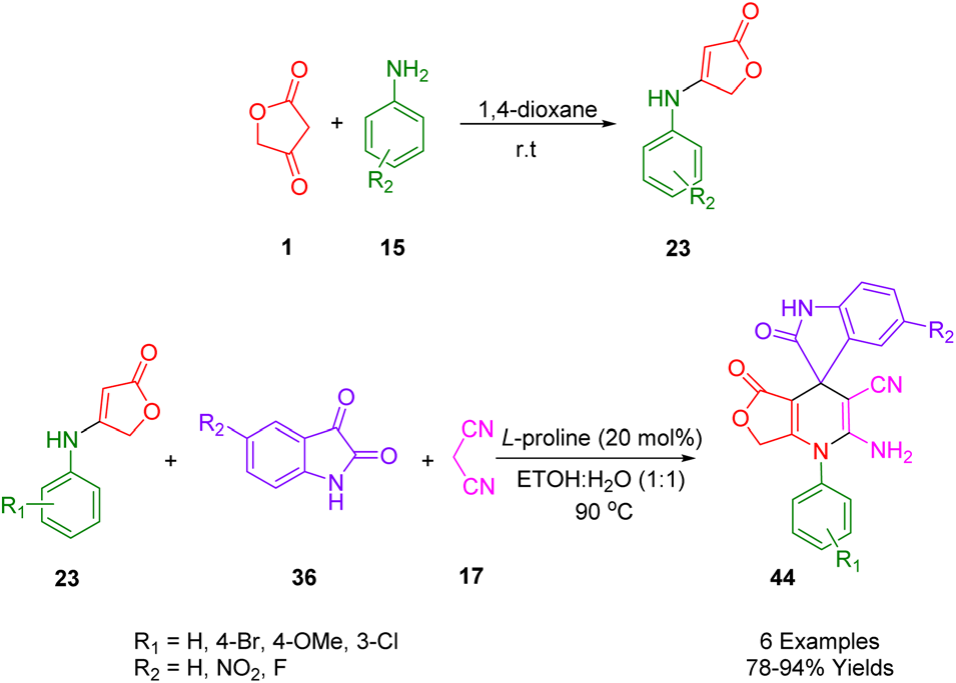
Synthesis of spirooxindole dihydropyridine derivatives.

Moradi *et al.*, reported an efficient and rapid strategy for the synthesis of the spirooxoindole dihydropyridine analogs (44) which was done with three component reactions including aldehydes (2), anilolactone (23) (to achieve the desired starting material, the reaction between tetronic acid (1) and substituted anilines (15) is used, and malononitrile (17) in aqueous ethanol solution (Scheme 21)). This protocol used 20 mol% of l-proline as an effective and cheap organocatalyst at 90 °C in EtOH/H_2_O (1 : 1) as a solvent and furnished the desired products in high yields.^41^

### 4.2. Benzoazulen-1-ones

**Scheme 22:**
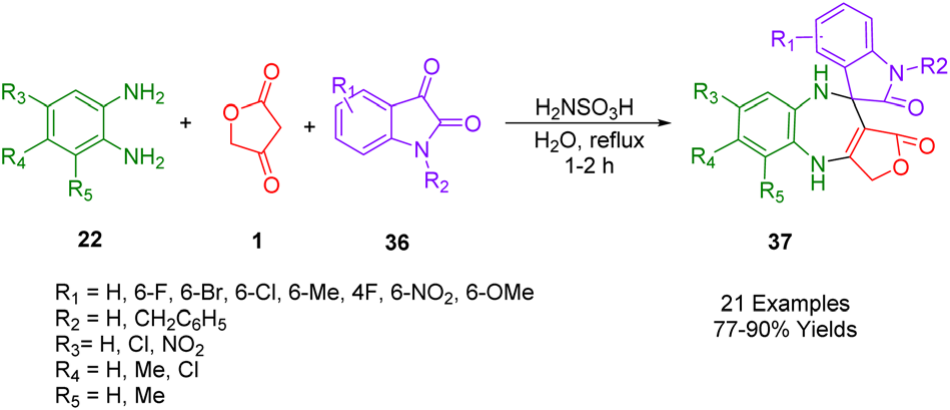
Synthesis of spirobenzodiazepines molecules.

Kamal *et al.*, demonstrated a simple, one-pot method of synthesizing spirobenzodiazepines (37) that is environmentally friendly as well. In the presence of mild and cheap catalysts such as sulfamic acid in refluxing water, tetronic acid (1), *o*-phenylenediamine (22), and isatins (36) can be synthesized in excellent yields through three-component reactions in water (Scheme 22). In this synthesis, there is no need for organic solvents that are harmful to the environment. Instead, water is used as the reaction medium, which can be separated by filtering, washing, and drying without further purification. Some cytotoxic properties of all the synthesized compounds have also been studied about their ability to kill different human cancer cell lines. Based on the results of the studies, it was determined that a majority of the compounds show cytotoxic activity ranging from moderate to effective.^52^

**Scheme 23:**
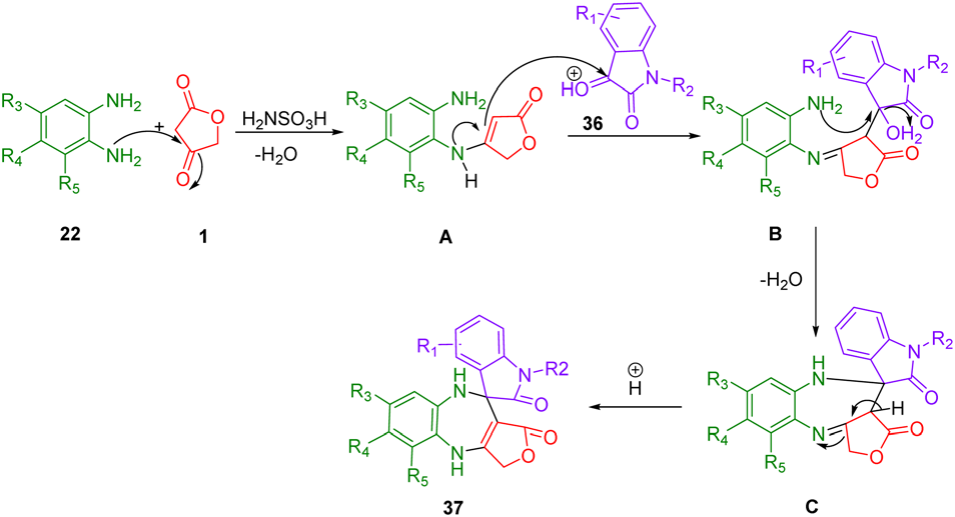
The plausible mechanistic approach for the synthesis of spiro-benzodiazepine.

According to Scheme 23, the synthesis of spirobenzodiazepines is proposed to proceed through the formation of two C–N bonds and one C–C bond, consistent with the suggested mechanism. Initially, intermediate A is generated as a result of the reaction between *o*-phenylenediamine (25) and tetronic acid (1). Subsequently, the enamine attacks the keto group of isatin (41), assisted by one amino group of *o*-phenylenediamine. This is followed by a second attack by another amino group from the enamine, resulting in a cyclization with isatin (41) to yield intermediate B. Following dehydration, intermediate B transforms into intermediate C. Ultimately, through intramolecular cyclization, intermediate C is converted into the final product, spiro benzodiazepine (43).

### 4.3. Spiro polyfunctionalized fused

**Scheme 24:**
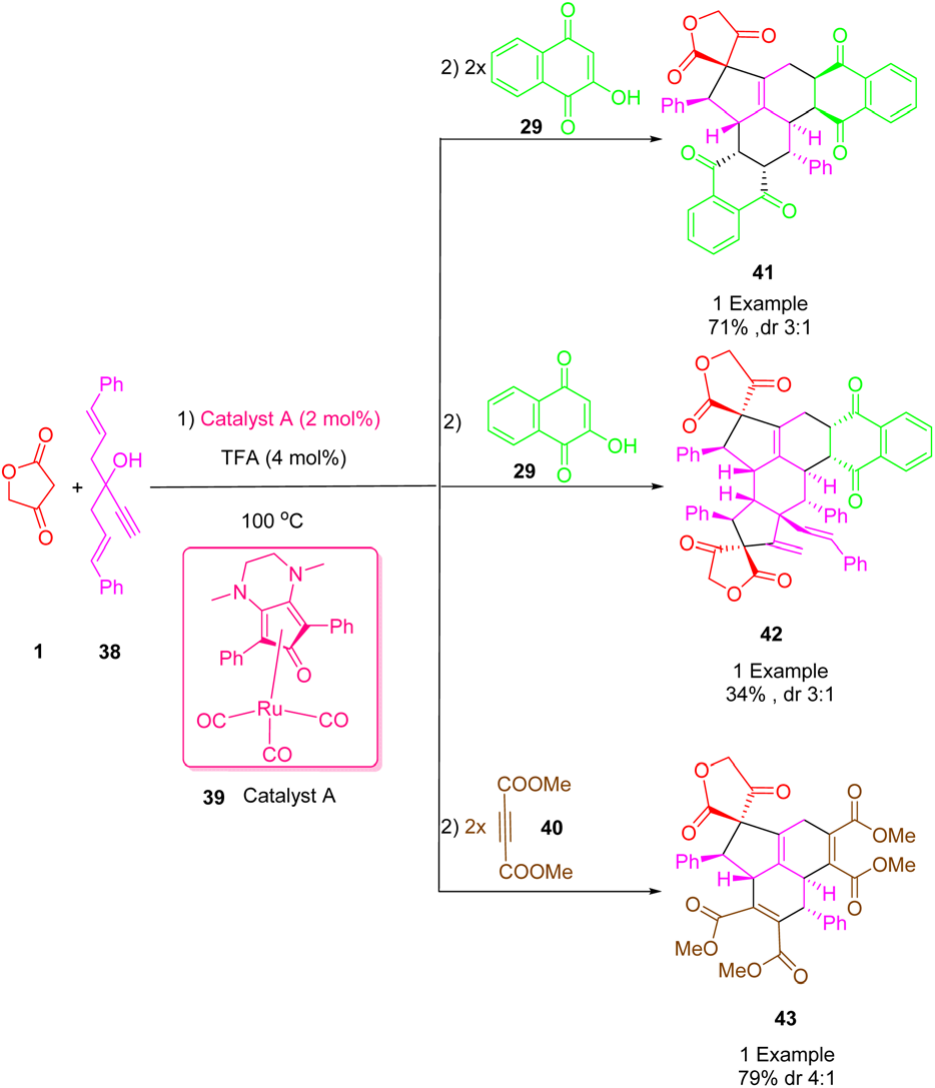
Synthesis of spiro highly functionalized fused polycycle cycloadducts.

One of the useful and practical tools in organic and pharmaceutical synthesis is the combination of one-pot protocols and multiple bond-forming cascade transformations. Using these atom-economic processes with simple precursors could provide rapid access to natural complex product-like molecules. An efficient transition-metal-catalyzed cascade reaction was described by Haak *et al.*, (Scheme 24) for the synthesis of biologically significant spiro highly functionalized fused polycycle cycloadducts (41), (42), (43) from the reaction between 1-alkenyl propargyl alcohols (3-ethynyl-1,5-di-phenylpenta-1,4-dien-3-ol) (38) with tetronic acid (1) and 1,4-naphthoquinone (29) or dimethyl acetylenedicarboxylate (DMAD) (40) respectively using bifunctional (cyclopentadienone) and ruthenium complexes of type A (39) as a catalyst in trifluoroacetic acid (TFA) in toluene at 100 °C with high diastereoselectivity yields. Therefore, it is possible to achieve a common process with high selectivity for multiple fused cycles of various structures and functionalized bicyclic structures. Meanwhile, the cytotoxic activity of some novel pseudo-pharmaceutical structures was observed against KB cells.^53^

## 5. Miscellaneous

**Scheme 25:**
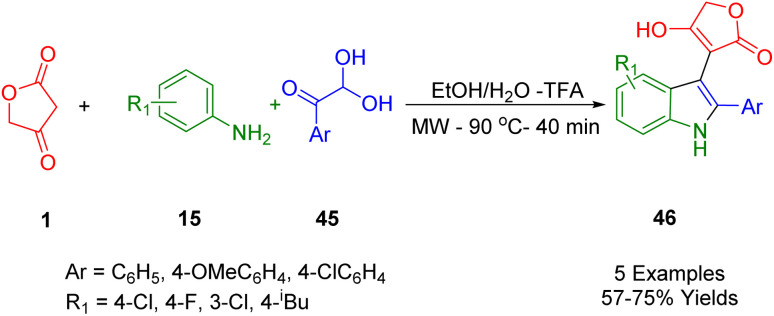
Synthesis of functionalized indoles.

Shi *et al.*, disclosed the microwave-assisted regioselective and facile synthesis of significant 3-functionalized indole compounds (46) *via* a domino reaction of tetronic acid (1) with arylglyoxal (45) and anilines (15) as starting materials in a solvent system containing ethanol and water (1 : 1) in trifluoroacetic acid (TFA) (Scheme 25). This method can be highly regarded due to its outstanding and remarkable advantages such as simplicity of practicality, short reaction time, green solvents, using non-metal, availability of raw materials, and high regioselectivity.^54^

**Scheme 26:**
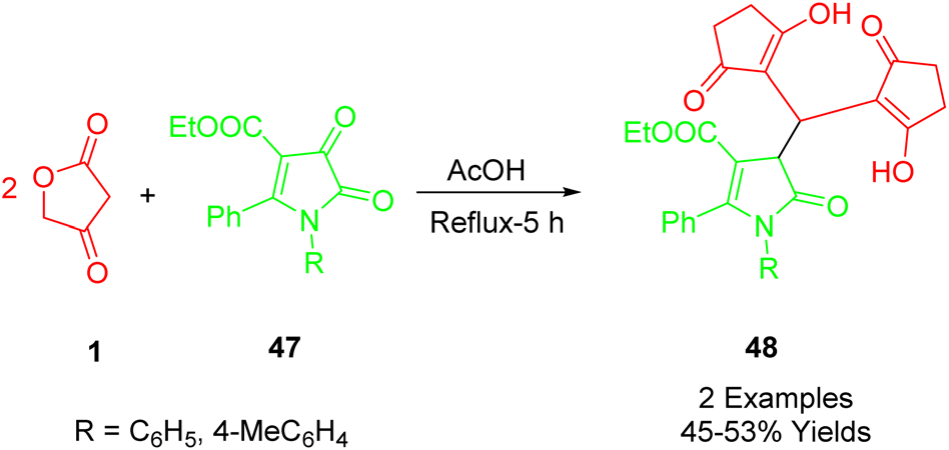
Synthesis of 1*H*-pyrrole-2,3-diones.

Maslivets *et al.*, synthesized ethyl 1-aryl-4,4-bis(4-hydroxy-2-oxo-2,5-dihydrofuran-3-yl)-5-oxo-2-phenyl-4,5-dihydro-1*H*-pyrrole-3-carboxylates (53) with favorable yields. This was accomplished using a straightforward, one-pot, three-component reaction. The process involved ethyl 1-aryl-4,5-dioxo-2-phenyl-4,5-dihydro-1*H*-pyrrole-3-carboxylates (52) and tetronic acid (1) in a 1 : 2 ratio. The reaction took place by boiling anhydrous toluene with acetic acid in dichloromethane (DCM) under reflux conditions (Scheme 26).^55^

**Scheme 27:**
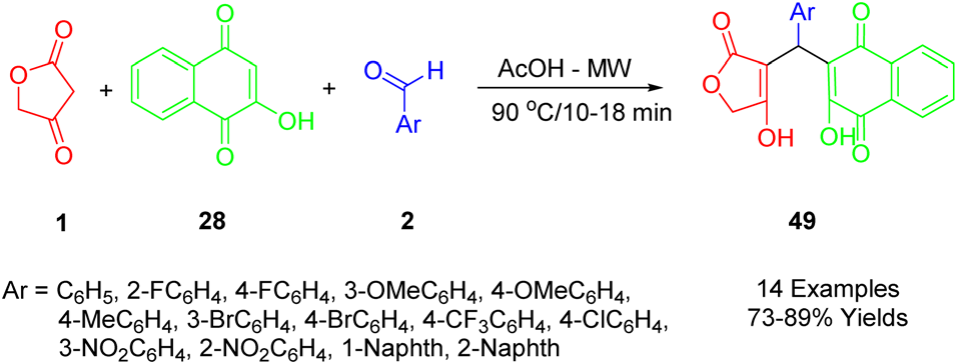
Synthesis of 3-arylated 2-hydroxynaphthalene-1,4-diones.

Nguyen used a microwave-assisted multicomponent reaction to prepare a series of novel 3-arylated 2-hydroxynaphthalene-1,4-dione compounds (54), employing tetronic acid (1), various aromatic aldehydes (2), and 2-hydroxynaphthalene-1,4-dione (37) (Scheme 27).^56^

Among the five synthetic compounds studied, half-maximal inhibitory concentrations were found to be approximately in the range of 4–20 nM against two human cancer cell lines, HepG2 and KB. The cytotoxicity of these synthesized compounds was evaluated in comparison to ellipticine, used as a positive control. The results showed that compounds with electron-donating groups were less toxic, and consequently less cytotoxic, than those with electron-withdrawing groups. It was observed that the cytotoxic activity of synthetic naphthoquinone compounds was influenced by the electronic properties of their substituents on the aromatic ring. In particular, the product with three fluoromethyl groups on its aromatic ring exhibited the highest IC50 against KB and HepG2 cancer cell lines, respectively, at 4.01 and 6.84 μM.

**Scheme 28:**
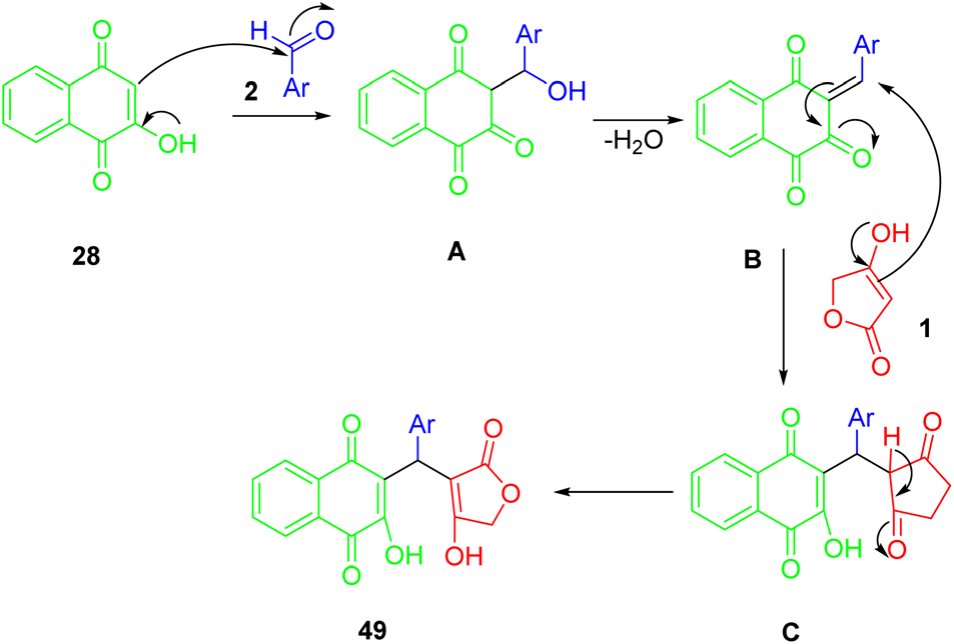
A plausible mechanism for the synthesis of 3-arylated 2-hydroxynaphthalene-1,4-diones.

According to Scheme 28, compounds (49) can be synthesized in the following way. A condensation of 2-hydroxynaphthalene-1,4-dione (28) with an aryl aldehyde (2) produces the intermediate A by Knoevenagel, which provides the conditions for the formation of arylidene ketone B after losing the water molecule from the intermediate. As a result of Michael's addition of tetronic acid (1) to intermediate B, this intermediate is deprotonated, and in the final step, it undergoes a 1,3-hydrogen shift to lead to the desired product (49).

## 6. Conclusions

Heterocycles, especially compounds with nitrogen or oxygen nuclei as their central structure, are of great interest in the development of compounds with biological and medicinal properties. Among compounds with dicarbonyl, tetronic acid derivatives can be used as an excellent starting material and a building block, and a key structural motif of natural compounds with different functional groups in the synthesis of heterocyclic frameworks. As a versatile synthon and a nucleus for the synthesis of complex heterocycles, MCRs of tetronic acid derivatives have recently made significant progress. MCR strategies have also consistently been proven to be a powerful method for synthesizing diverse and complex molecular systems. Research on tetronic acid's uses in multicomponent reactions has been summarized in this review. This helped us synthesize a variety of polyfunctional heterocyclic scaffolds between 2017 and 2022.

## Conflicts of interest

There are no conflicts to declare.

## Supplementary Material
